# Inferences of evolutionary history of a widely distributed mangrove species, *Bruguiera gymnorrhiza*, in the Indo-West Pacific region

**DOI:** 10.1002/ece3.624

**Published:** 2013-06-07

**Authors:** Chie Urashi, Kosuke M Teshima, Sumiko Minobe, Osamu Koizumi, Nobuyuki Inomata

**Affiliations:** 1 Department of Environmental Science, International College of Arts and Sciences, Fukuoka Women's University1-1-1 Kasumigaoka, Higashi-ku, Fukuoka, 813-8529, Japan; 2 Department of Biology, Faculty of Sciences, Kyushu University6-10-1, Hakozaki, Higashi-ku, Fukuoka, 812-8581, Japan

**Keywords:** Demographic history, DNA sequence variation, geographical or land barrier, mangrove, population structure

## Abstract

Inference of genetic structure and demographic history is fundamental issue in evolutionary biology. We examined the levels and patterns of genetic variation of a widespread mangrove species in the Indo-West Pacific region, *Bruguiera gymnorrhiza*, using ten nuclear gene regions. Genetic variation of individual populations covering its distribution range was low, but as the entire species it was comparable to other plant species. Genetic differentiation among the investigated populations was high. They could be divided into two genetic clusters: the West and East clusters of the Malay Peninsula. Our results indicated that these two genetic clusters derived from their ancestral population whose effective size of which was much larger compared to the two extant clusters. The point estimate of speciation time between *B. gymnorrhiza* and *Bruguiera sexangula* was two times older than that of divergence time between the two clusters. Migration from the West cluster to the East cluster was much higher than the opposite direction but both estimated migration rates were low. The past Sundaland and/or the present Malay Peninsula are likely to prevent gene flow between the West and East clusters and function as a geographical or land barrier.

## Introduction

Mangrove species constitute a unique ecosystem in coastal zones of tropical and subtropical regions. The distribution of mangrove species is classified into two major regions, Indo-West Pacific (IWP) and Atlantic-East Pacific (AEP) (Tomlinson 1986; Duke et al. 1998). The IWP has about four times greater species diversity than the AEP (Duke et al. 1998). In the last few decades, many mangrove forests are rapidly getting small, fragmented, and lost in both regions (e.g., FAO 2007). Population size reduction and fragmentation cause a loss of genetic diversity and lead to the loss of adaptability for environmental changes. The loss of adaptability increases the extinction risk. For successive preservation and management of this unique ecosystem, the accurate assessment of genetic diversity and population genetic structure of mangrove species is essential. The knowledge of genetic information also provides us a perspective of evolutionary mechanisms that shaped current biodiversity and adaptability, and gives us insight how to restore this unique ecosystem.

Most studies on genetic variability and population structure of mangrove species use simple sequence repeat (SSR) markers and chloroplast DNA (cpDNA) sequences (e.g., Marguire et al. 2000; Liao et al. 2007; Huang et al. 2008; Jian et al. 2010; Pil et al. 2011). Polymorphic SSR loci are “selected” as markers in population genetic studies. This selection leads to the overestimation of the amount of genetic variation in a population. In addition, it is not easy to apply SSR markers for one species to the other species. In contrast, surveys of genetic structure on mangroves using nucleotide sequences are very limited (Zhou et al. 2008; Inomata et al. 2009; Minobe et al. 2010) because nucleotide sequence variation in mangrove species is considered to be low and, therefore, not useful for population genetic studies. However, surveys on gene sequence variation are crucial to find evolutionary forces that shaped genetic structures and to identity genes that are responsible for adaptation. For example, Zhou et al. (2011) reported some candidate genes for local adaptation based on the survey of 71 genes in a mangrove species, *Sonneratia alba*. To proceed future comparative evolutionary genomics studies, accumulation of gene sequence data is indispensable. Furthermore, for most mangrove species, assessment of genetic structure of populations was mainly focused on limited areas (e.g., Inomata et al. 2009). To draw a general picture of genetic structure and evolutionary history, surveys of populations covering areas of species distribution are necessary.

Mating systems and the ways of pollen and seed dispersal are important factors affecting genetic structure of plant populations. Dispersal distance of pollen and seeds is one of the determinants for geographical distribution range of plant species. Previous studies on mangrove species revealed that, in general, genetic variation within populations is low and genetic differentiation among populations is high (e.g., Goodall and Stoddart 1989; Giang et al. 2006; Liao et al. 2007; Inomata et al. 2009; Minobe et al. 2010; Pil et al. 2011). High genetic differentiation indicates low gene flow between populations, suggesting pollen and seed dispersal are restricted. Indeed, propagules of *Rhizophora mucronata* and *Rhizophora apiculata* appear to have short dispersal ability (Drexler 2001) and the average distances of pollen and propagule dispersal are also short in *Kandelia candel* (Geng et al. 2008).

Plants with sea-drifted seeds and animals living in water cannot migrate through land. In general, this is called a geographical or land barrier hypothesis. In the IWP region, the sea level dropped to 100–120 m below the current level during the Last Glacial Maximum (LGM) (Voris 2000) and thus the Sundaland, which consists of the Malay Peninsula, Sumatra, Java, Borneo islands, and exposed shelves, existed at that time. During the LGM, the Sundaland is considered to have played a role as a land barrier that prevented gene flow between the Pacific and Indian Oceans. At present, the Malay Peninsula is suggested to continue to serve as a barrier to gene flow between the Pacific and Indian Oceans. Because propagules of all mangroves are dispersed only by water (Tomlinson 1986), past and current land barriers are considered to affect genetic structure of the extant mangrove populations (Liao et al. 2007; Huang et al. 2008; Inomata et al. 2009).

Our main objective is to clarify genetic structure and demographic history of typical mangrove species on a global scale. For this purpose, we examined genetic variation of ten nuclear gene regions as well as one cpDNA region using samples from ten *Bruguiera gymnorrhiza* populations covering its natural distribution range. *B. gymnorrhiza* (L.) Lamk. (Rhizophoraceae) is a typical mangrove species and one of the predominant mangrove species in the IWP region. It has the broadest distribution in the genus: from eastern coast of Africa in the west through Asia, northward to the Ryukyu Islands of southern Japan and southward in tropical Australia (Tomlinson 1986). It can be found in the middle and upper intertidal zones. Like the other mangrove species of the tribe Rhizophoraceae Blume, *B. gymnorrhiza* has a unique viviparous propagule (Tomlinson 1986), which is dispersed by water. *B. gymnorrhiza* is one of the bird-pollinated large-flowered species of *Bruguiera* (Tomlinson 1986). For interspecies comparison, genetic variation of the identical gene regions of the closely related species, *Bruguiera sexangula* (Lour.) Poir., was also examined.

We address the following specific questions: (1) What is the extent of nucleotide sequence variation? (2) What is the extent of genetic differentiation between populations? (3) Do the Sundaland and/or the Malay Peninsula play a role of a barrier to prevent gene flow between the Pacific and Indian Oceans? (4) When did the two species diverge?

## Materials and Methods

### Plant samples

**Figure 1:**
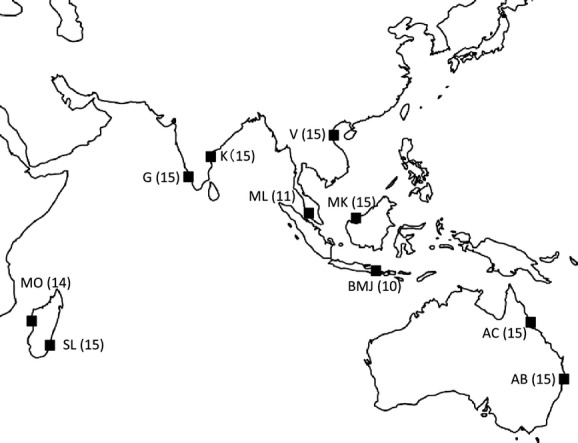
Sampling locations of the 10 populations. Populations are indicated by abbreviations. Number of individuals used in this study is shown in parentheses.

Leaf materials of *B. gymnorrhiza* were sampled from different individuals at the following ten locations (Fig. 1); V (Hanoi, Vietnam) located in the east of the Asian Continent, MK (Kuching, Malaysia) located in the northwest of the Borneo Island in the Southeast Asia, ML (Langkawi Island, Malaysia) located in the northwest side of the Malay Peninsula, BMJ (Bali Island, Indonesia) located in the east of the Java Island in the Southeast Asia, AC (Cairns, Australia) located in the northeast coast of Australia, AB (Ballina, Australia) located in the east coast of Australia, K (Kakinada, India) located in the northeast coast of India, G (Goa, India) located in the southwest of India, SL (Tolanaro, Madagascar) located in the southeast coast of Madagascar, and MO (Morondava, Madagascar) located in the west coast of Madagascar. We call each sampling location as a population in the following sections. Leaf materials of a closely related species, *B. sexangula*, were also sampled at two locations on the western coast of the Malay Peninsula. The details of our samples, including the locations and numbers of individuals, are summarized in Table 1.

**Table 1:** Geographical location and sample size in *Bruguiera gymnorrhiza* and *Bruguiera sexangula*

Sampling location	Abbreviation	Geographical coordinates	Number of individuals
*B. gymnorrhiza*			
Australia			
Ballina	AB	28°52′33.26″S, 153°32′47.05″E	15 (55)
Cairns	AC	16°50′21.47″S, 145°43′25.45″E	15 (32)
Indonesia			
Bali	BMJ	8°05′45.84″S, 114°28′33.15″E	10 (10)
Vietnam			
Hanoi	V	21°00′05.62″N, 107°16′35.82″E	15 (45)
Malaysia			
Kuching	MK	1°38′31.22″N, 110°14′57.25″E	15 (45)
Langkawi	ML	6°26′02.61″N, 99°50′23.41″E	11 (11)
India			
Goa	G	15°22′23.85″N, 73°57′42.07″E	15 (23)
Kakinada	K	16°56′48.14″N, 82°15′23.12″E	15 (20)
Madagascar			
Morondava	MO	20°16′47.58″S, 44°17′19.81″E	14 (41)
Tolanaro	SL	25°03′43.35″S, 46°53′42.07″E	15 (29)
*B. sexangula*			
Malaysia			
Langkawi		6°26′02.61″N, 99°50′23.41″E	7 (8)
Thailand			
Ranong		9°84′43.92″N, 98°55′40.58″E	2 (2)

Number of individuals used in this study is shown. Total number of individuals collected is shown in parentheses.

### DNA extraction, PCR, and sequencing

Fifteen individuals were randomly selected from each population. When the number of individuals in a population was smaller than 15, we analyzed all individuals. Genomic DNA was extracted from a piece of a single leaf using modified cetyltrimethylammonium bromide (CTAB) method (Murray and Tompson 1980). In total, genomic DNAs of 140 individuals of *B. gymnorrhiza* were obtained.

Based on the published sequence information of the expressed sequence tags (ESTs) of *B. gymnorrhiza* (Miyama et al. 2006), PCR primer sets of eight nuclear genes were designed. Based on the sequence information of the PCR products, we conducted BLAST search against NCBI database (nucleotide collection). According to the blast search results, we tentatively named the eight genes as follows; *NAC* (BP941102), *VVHP* (BP944085), *PO* (BP939063), *GM* (BP939421), *SF* (BP939928), *EXP2* (BP942725), *EPCRF* (BP946081), and *UNK* (BP945250), where the corresponding *B. gymnorrhiza* ESTs (Miyama et al. 2006) are shown in parentheses. In addition to the aforementioned eight nuclear genes, two nuclear genes, *PAL*1 and *mang*-1 (Inomata et al. 2009), and one chloroplast DNA (cpDNA) region, *trn*S-*trn*G spacer (Hamilton 1999; Sun and Lo 2011), were also analyzed.

PCR amplification was carried out under the following protocols: an initial denaturation at 95°C for 3 min followed by 30 cycles of denaturation at 95°C for 30 sec, annealing at 50°C, 55°C, or 60°C for 30 sec, polymerization at 72°C for 1.5 min, and a final extension at 72°C for 7 min. The details of the sequence and annealing temperature of PCR primers are listed in Table S1. After the amplification, PCR products were purified. Then we directly determined their sequences using a DNA sequencing kit (BigDye terminator v. 3.1/1.1 cycle sequencing kit, ABI) with the PCR primers and ABI Prism 3100 or 3730 automatic sequencers (Applied Biosystems, Tokyo, Japan).

Two haplotype sequences were determined for each individual. After the direct sequencing when we found no or only one heterozygous site in a sequence of a single individual, we inferred sequences of two haplotypes for each individual. On the other hand, when two or more heterozygous sites or indels were found in a sequence of a single individual, purified PCR product was cloned into the pGEM T-easy vector (Promega KK, Tokyo, Japan). Then, we sequenced each clone using T7 and Reverse primers designed at the promoter sites of the vector (Table S1). Four to sixteen clones were sequenced for each individual. Sequences of two haplotypes of a heterozygous individual were determined when we obtained at least two exactly identical sequences for each haplotype.

### Data analyses

Sequences of each gene were aligned using CLUSTAL X 2.1 (Larkin et al. 2007) and further edited by hand. Because, we could not amplify *EXP2* gene of one individual sampled from the AB population from the east coast of Australia, we treated it as missing data in the following analysis. A continuous alignment gap was treated as a single indel. All sequences obtained in this study were deposited in the DNA Data Bank of Japan (DDBJ). Accession numbers are AB813916-AB817042.

Molecular population genetic analysis of *B. gymnorrhiza* was conducted using sequences of *B. sexangula* as an outgroup. Using inter-simple sequence repeat, ribosomal internal transcribed spacer, and chloroplast DNA markers, Sun and Lo (2011) showed that *B. gymnorrhiza* and *B. sexangula* are genetically close relatives. Molecular population genetic parameters, *π* (the average number of nucleotide differences per site: nucleotide diversity; Nei 1987), *K*_*s*_ (number of nucleotide substitutions per silent site between species), and *K*_*a*_ (number of nucleotide substitutions per replacement site between species), were estimated using the DnaSP ver. 5.10.00 (Librado and Rozas 2009). To assess the deviation from the standard neutral model, Tajima's test, Fu and Li's test, Fay and Fu's test, and MK test were performed using the DnaSP version 5.10.00, and the multilocus HKA test was also performed using HKA program downloaded from http://genfaculty.rutgers.edu/hey/software#HKA.

The extent of genetic differentiation among populations was estimated by analysis of molecular variance (AMOVA) approach (e.g., Weir and Cockerham 1984). The Arlequin software version 3.5.1.3 (Excoffier and Lischer 2010) was used to estimate *F*-statistics. Three hierarchical levels, (i) among populations, (ii) among individuals/within populations, and (iii) within individuals, were examined for nuclear genes by the locus-by-locus analysis, and two levels, (i) among populations and (ii) within populations, were examined for cpDNA. Each haplotype in a gene was regarded as an allele. Overall values of *F*-statistics for all the ten nuclear genes were obtained by summing variance components over the genes. In the AMOVA analyses, genotypic data of nuclear genes, where gametic phase of genotype is unknown, and haplotypic data of the cpDNA region were used. A haplotype network of the cpDNA region was constructed using the TCS ver. 1.21 (Clement et al. 2000).

Genetic clusters of populations were inferred using the Bayesian clustering approach, STRUCTURE ver. 2.3.3 (Pritchard et al. 2000; Falush et al. 2003; Hubisz et al. 2009). Under the admixture model, five independent runs were performed for each number of clusters (*K*: *K* = 1–10) to confirm the convergence of Markov chain Monte Carlo (MCMC) chains. In each run, 500,000 MCMC iterations were performed after a burn-in period of 1,000,000 iterations. To estimate the number of clusters, we applied the method proposed by Evanno et al. (2005); *K* is identified when delta *K*, which is the rate of change in the log probability of data between continuous *K* values, is maximal.

Population history of genetic clusters identified using the STRUCTURE and evolutionary history of the two species, *B. gymnorrhiza* and *B. sexangula*, were inferred. Population parameters were estimated under the isolation-with-migration (IM) model using the IMa2 program (Hey and Nielsen 2007; Hey 2010). In this analysis non-recombinant regions were used. Non-recombinant regions were estimated using the four-gametic test (Hudson and Kaplan 1985) implemented in DnaSp ver. 5.10.00. Population splitting (or speciation) times (*T* = *tu*), population sizes (*θ* = 4*Nu*), and migration rates (*2Nm*) between genetic clusters (or species) were estimated, where *u* is the mutation rate per locus per generation, *t* is the population splitting (or speciation) time, *N* is the effective population size, and *m* is the migration rate. The infinite site model (Kimura 1969) was used except *VVHP*, *EXP*2, and *mang*-1. For the three regions, we applied HKY model (Hasegawa et al. 1985) because there were more than two alleles at some variable sites in these three genes. The upper bounds of the parameters are set to include most part of the posterior distribution of preliminary simulations. We confirmed that the burn-in period was long enough so that the MCMC simulation reached the stationary state. Each simulation run was performed with 40 chains with adjusted heating terms according to the IMa2 manual. To check the convergence of the MCMC simulation to the true stationary state, multiple independent runs were performed. The posterior distributions of parameters were estimated using 100,000 genealogies sampled from multiple independent runs. Peaks of the posterior distribution were defined as estimates of parameters.

## Results

### Nucleotide variation in *B. gymnorrhiza*

Sequences of partial regions of the ten nuclear genes, *NAC*, *VVHP*, *PO*, *GM*, *SF*, *EXP*2, *UNK*, *EPCRF*, *PAL*1, and *mang-*1, were determined for ten populations of *B. gymnorrhiza*. Sequences of one cpDNA region, *trnS-trnG* spacer, were also determined. The levels of DNA variation in *B. gymnorrhiza* are summarized in Table 2. In *B. gymnorrhiza* total length examined for nuclear genes was 5,295 bp including alignment gaps, where 3,313 bp was coding region. In total 84 segregating sites and five indels were found. In 581 bp of the cpDNA region, there were three segregating sites and five indels.

**Table 2:** Summary of nucleotide polymorphism in *Bruguiera gymnorrhiza*

	Alignment length/Number of silent sites (bp)	*n*	*S*	Indel	*π*_*t*_	*π*_*s*_	*π*_*a*_
Nuclear gene							
*NAC*	370/185.17	280	0	0	0.00	0.00	0.00
*VVHP*	575/381.06	280	26 (22/4)	2	9.06	11.89	3.29
*PO*	536/237.67	280	0	0	0.00	0.00	0.00
*GM*	494/282.50	280	9 (7/2)	0	6.57	11.51	0.07
*SF*	407/100.01	280	3 (1/2)	0	2.17	8.90	0.00
*EXP*2	535/433.89	278	18 (18/0)	1	13.12	16.21	0.00
*UNK*	426/102.33	280	2 (0/2)	0	1.03	0.00	1.35
*EPCRF*	408/82.75	280	7 (4/3)	0	5.02	25.06	0.09
*PAL*1	704/169.66	280	4 (2/2)	2	1.30	3.27	0.68
*mang-*1	840/773.67	280	15 (15/0)	0	2.87	3.12	0.00
Average	530/274.87	279.8	8.4 (6.9/1.5)	0.5	4.11	8.00	0.55
cpDNA region							
*trnS-trnG*	581/506.00	140	3	5	0.08	0.08	0.00

Alignment length/Number of silent sites, Sequence length includes alignment gaps. Number of silent sites does not include alignment gaps. *n*, number of sequences; *S*, Number of segregating sites excluding indels. Numbers of silent/replacement differences are shown in parentheses; Indel, number of indels. Continuous alignment gap was counted as a single indel; *π*_*t*_, number of nucleotide differences per total site (Nucleotide diversity; Nei 1987) with the Jukes and Cantor (1969) correction. Indels are not included. The value was multiplied by 10^3^; *π*_*s*_, number of nucleotide differences per silent site with the Jukes and Cantor (1969) correction. Indels are not included. The value was multiplied by 10^3^; *π*_*a*_, number of nucleotide differences per nonsynonymous site with the Jukes and Cantor (1969) correction. Indels are not included. The value was multiplied by 10^3^.

The level of nucleotide variation was different among the genes. For example, nucleotide diversity (Nei 1987) per silent site, *π*_*s*_, for the total population ranged from 0 (*NAC* and *PO*) to 0.02506 (*EPCRF*) for the nuclear genes (Table 2). Two of the ten genes (*NAC* and *PO*) were monomorphic. In the total population, the average *π*_*s*_ over the ten nuclear genes was 0.00800. The *π*_*s*_ value of the cpDNA region was 0.00008. Nucleotide diversity of the nuclear genes was ∼100 times higher than that of the cpDNA region, indicating that the nuclear genes are more useful markers than the cpDNA region.

The level of nucleotide variation also varied among the ten populations. For example, the average *π*_*s*_ value over the ten nuclear genes ranged from 0 (G and K populations from the southwest and northeast India) to 0.00424 (V population from the east of the Asia Continent) (Table S2).

### Nucleotide divergence between *B. gymnorrhiza* and *B. sexangula*

Nucleotide divergence between *B. gymnorrhiza* and *B. sexangula* is summarized in Table 3. No shared polymorphism was found between the two species. The number of fixed nucleotide substitutions ranged from 1 to 11 in the nuclear genes. On average, there were 3.9 fixed nucleotide substitutions between the two species. These results confirm that our sample consists of two distinct species. The number of nucleotide substitutions per silent site between the species (*K*_*s*_) ranged from 0.00437 to 0.04925 for the nuclear genes and 0.00334 for the cpDNA region. The average *K*_*s*_ value over the ten nuclear genes was 0.02162. Divergence at silent sites of nuclear genes was ∼10 times higher than that of the cpDNA region. The number of nucleotide substitution per replacement site between the species (*K*_*a*_) ranged from 0.00000 to 0.00485 for the nuclear genes. The highest value of *K*_*a*_/*K*_*s*_ was 0.32071 for *PAL*1, indicating that these nuclear genes are not pseudogenes.

**Table 3:** Summary of nucleotide divergence between *Bruguiera gymnorrhiza* and *Bruguiera sexangula*

	Length (including gaps)	*n*	Fix	Indel	*K*_*s*_	*K*_*a*_	*K*_*a*_/*K*_*s*_
Nuclear gene							
*NAC*	370	298	4 (2/2)	0	21.92	0.00	0.00
*VVHP*	575	298	5 (5/0)	1	23.25	2.51	0.11
*PO*	536	298	1 (1/0)	1	4.37	0.00	0.00
*GM*	495	298	4 (3/1)	1	22.21	4.85	0.22
*SF*	407	298	4 (0/4)	0	49.25	0.00	0.00
*EXP*2	538	296	4 (4/0)	1	25.70	0.00	0.00
*UNK*	426	298	3 (2/1)	0	19.81	4.04	0.20
*EPCRF*	408	298	1 (0/1)	0	24.56	3.13	0.13
*PAL*1	704	298	2 (1/1)	0	7.92	2.54	0.32
*mang*-1	842	298	11 (11/0)	2	17.23	0.00	0.00
Average	530	297.8	3.9	0.6	21.62	1.71	0.10
cpDNA region							
*trn*S-*trn*G	581	149	2	0	3.34	0.00	0.00

Length, Sequence length includes alignment gaps. *n*, number of sequences; Fix, number of fixed nucleotide differences between the species excluding indels. Numbers of silent/replacement differences are shown in parentheses; Indel, number of fixed indels between the species. A continuous gap was counted as a single indel; *K*_*s*_, number of nucleotide substitutions per silent site with the Jukes and Cantor (1969) correction. Indels are not included. The value was multiplied by 10^3^; *K*_*a*_, number of nucleotide substitution per replacement site with the Jukes and Cantor (1969) correction. Indels are not included. The value was multiplied by 10^3^.

### Neutrality tests

Results of neutrality tests are summarized in Table S3. Most results of Tajima's test and Fu and Li's test were not significant. For example, Tajima's *D* values were not significant except for the *VVHP* gene of MO population (the west of Madagascar) and the *mang*-1 gene of the V population (the eastern part of the Asian Continent). No significant results were obtained in Fay and Fu's test (Table S3). The result of the multilocus HKA test was weakly significant (*P* = 0.048). In MK test no significant result was found (data not shown). These results suggest that gene regions examined could be treated as neutral markers in *B. gymnorrhiza*.

### Genetic structure of populations

*F*-statistics among the ten populations estimated by the AMOVA approach were shown in Table 4. Two of the ten genes (*NAC* and *PO*) had no variation, and therefore they did not have any information for population structure. The estimated *F*_*ST*_ values were highly significant both for nuclear genes and cpDNA region, indicating that there is genetic differentiation among the ten populations. In nuclear genes most population pairwise *F*_*ST*_ values were highly significant except for population pairs, AB (eastern Australia) – MK (northwestern Borneo), AB – ML (northwestern part of the Malay Peninsula), AC (northeastern of Australia) – ML, BMJ (eastern Java) – MO (western Madagascar), V (eastern part of Asian Continent) – K (northeastern India), MK – ML, and G (southwestern India) – SL (southeastern Madagascar) (Table S4), indicating that most population pairs are genetically differentiated. Although the pairwise *F*_*ST*_ values in nuclear genes were not significant for population pairs, AB (eastern Australia) – MK (northwestern Borneo), AB – ML (northwestern part of the Malay Peninsula), and AC (northeastern Australia) – ML, a relationship of cpDNA haplotypes (Figure S2) and geographic distribution of the haplotypes (Figure S3) are likely to indicate genetic differentiation between the Australian populations and others.

**Table 4:** *F*-statistics among the ten populations

Nuclear genes (overall 10 loci)		

*F*_*IS*_	*F*_*ST*_	*F*_*IT*_
0.29192 (≪0.001)	0.78638 (≪0.001)	0.84874 (≪0.001)

cpDNA region		

*F*_*IS*_	*F*_*ST*_	*F*_*IT*_

na	0.88932 (≪0.001)	na

*P-*values are showed in parentheses; na, not applicable.

**Figure 2:**
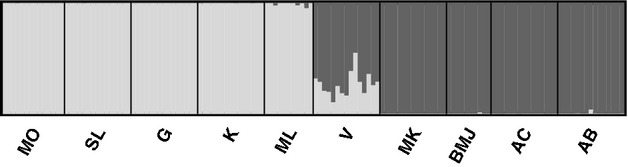
Clustering results of individuals in the 10 popultions of *Bruguiera gymnorrhiza* were estimated using the STRUCTURE. Each vertical bar in the histogram represents the proportion of cluster memberships in a single individual. Abbreviations of populations ordered from the west to the east are shown under the histogram.

To find the uppermost hierarchical level of the genetic clusters, population structure was estimated using the STRUCTURE program (Pritchard et al. 2000; Hubisz et al. 2009). It assigns individuals into *K* clusters, each of which is at HWE or not in LD, using multilocus genotype data. According to the method of Evanno et al. (2005), the number of clusters was estimated to be 2 (Figure S1). The result of the STRUCTURE analysis is shown in Figure 2. The first cluster (dark gray) includes two Australian populations (AB and AC; see Figure 1) and three populations located on the east coast of the Malay Peninsula (BMJ, V, and MK; see Fig. 1) (East cluster). The second cluster (light gray) includes five populations located on the west coast of the Malay Peninsula (ML, G, K, MO, and SL; see Fig. 1) (West cluster).

### Population history

**Figure 3:**
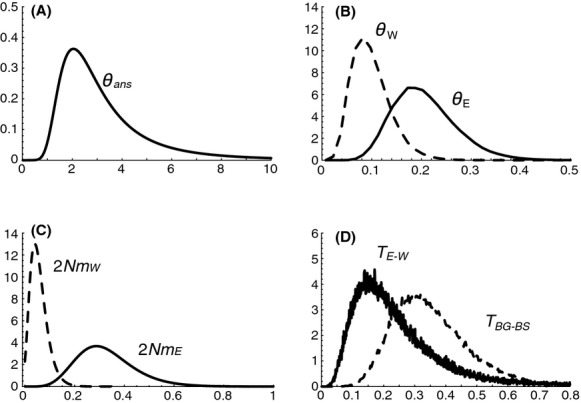
Marginal posterior probability distributions for model parameters estimated using the IMa2. (A) Population size of Ancestor population (*θ*_*ans*_). (B) Population size of the East cluster (*θ*_*E*_) and West cluster (*θ*_*W*_). (C) Migration rate from the West cluster (2*Nm*_*E*_) to the East cluster and from the East cluster to the West cluster (2*Nm*_*W*_). (D) Divergence time of the East cluster and West cluster (*T*_*E−W*_) and speciation time between *Bruguiera gymnorrhiza* and *Bruguiera sexangula* (*T*_*BG−BS*_).

To infer the population history of the two genetic clusters identified by the STRUCTURE analysis, population parameters were estimated under the IM model using IMa2 program (Hey and Nielsen 2007; Hey 2010). Evolutionary history of the two investigated species was also inferred (Fig. 3). The estimated splitting time between the two population clusters was 0.17 (95% highest posterior density (HPD), lower bound: 0.04, upper bound: 0.54) in units of mutation time scale (time x mutation rate), whereas the estimated speciation time between *B. gymnorrhiza* and *B. sexangula* was 0.32 (95% HPD, lower bound: 0.13, upper bound: 0.63). This result indicates the speciation event between *B. gymnorrhiza* and *B. sexangula* is roughly two times older than the divergence of the two clusters in *B. gymnorrhiza*.

The estimate of *θ* for the East cluster (*θ*_*E*_) was 0.17 (95% HPD, lower bound: 0.08, upper bound: 0.38) and *θ* for the West cluster (*θ*_*W*_) was 0.08 (95% HPD, lower bound: 0.04, upper bound: 0.26). *θ* for the ancestral population was 2.05 (95% HPD, lower bound: 0.82, upper bound: 7.37). This result means that the effective population size of East cluster is larger than that of West cluster, and the ancestral population is much larger than either of the clusters. Larger size in ancestral populations than the extant populations was also reported in the other mangrove species (*Sonneratia*, Zhou et al. 2007). There was more migration from the West cluster to the East cluster (2*Nm*_*E*_ = 0.29 [95% HPD, lower bound: 0.12, upper bound: 0.56]) than from the East to West (2*Nm*_*W*_ = 0.04 [95% HPD, lower bound: 0, upper bound: 0.12]), although in both cases the migration was rare (2*Nm* < 1). No migration was estimated between *B. gymnorrhiza* and *B. sexangula* after the speciation event (data not shown).

## Discussion

### DNA polymorphism and divergence

To find a general picture of levels and patterns of DNA variation of a broad range species, *B. gymnorrhiza*, leaf materials were collected from 10 localities covering its distribution range and DNA sequences were determined. General pattern of nucleotide variation of *B. gymnorrhiza* was as follows: (1) low polymorphism in each local population but (2) heterogeneous variation between the local populations (Table S2). As the entire species, however, the level of polymorphism was comparable to other plant species (e.g., *Cryptomeria japonica*; Kado et al. 2003; *Arabidopsis thaliana*; Schmid et al. 2005; *Pinus* species; Ma et al. 2006; see summarized by Gossmann et al. 2010). Assuming neutral evolution of the investigated gene regions, the simplest interpretation of the result is that the effective population sizes of the local populations are different. Even though the number of individuals is large in nature, effective size of some populations could be quite small.

In each population the level of nucleotide variation was low. For example, in Indian populations, G and K, no polymorphism was found for all the ten genes. Inbreeding is one of the plausible explanations. Other plausible explanation is that originally each local population may have been founded by a small number of individuals and not enough time has elapsed to accumulate new mutations. The bottleneck and/or founder effects caused by the climatic oscillations and environmental changes during the Pleistocene are also possible explanations for the low level of nucleotide variation in local populations. These do not rule out the possibility of local adaptation. In any cases if low genetic variation is general feature in functional (or protein-coding) genes, each population is likely to have little potential for genetic adaptability. This suggests that extinction risk of each population is high. Alternatively, it may be the nature of this species, for example, the species may repeat the following extinction-recolonization cycle; (1) a population starts with a small number of individuals, which adapted (or survived) to a given environment, (2) the population increase in size (number of individuals), and (3) when the environment changes accompanying with climatic and/or geographical changes, the population disappears.

Nucleotide divergence at silent sites between *B. gymnorrhiza* and *B. sexangula* was ∼2%. It was similar or smaller that nucleotide divergence between *Sonneratia* species (2.06 ∼ 3.75%, Zhou et al. 2007) and between *R. apiculata* and *R. mucronata* (∼2.7%, Inomata et al. 2009). On the other hand, as the entire species, nucleotide diversity at silent sites in *B. gymnorrhiza* (0.00800) was comparable to those of *Sonneratia alba* (0.00432) and *S. caseolaris* (0.01003) (Zhou et al. 2007), but two order higher than those of *R. apiculata* (0.00059) and *R. mucronata* (0.00003) (Inomata et al. 2009). Like in this study, population samples of the two *Sonneratia* species were collected from across the entire IWP region. However, in the previous study (Inomata et al. 2009), the two *Rhizophora* species were collected from only three localities in Thailand, which are a part of their distribution range in the IWP region. Wider range of samples in the two *Rhizophora* species shows higher polymorphism than the previous study (W. L. Ng, N. Inomata, K. M. Teshima, S. Changtragoon, I. Z. Siregar, A. E. Szmidt, unpubl. data). Similar trend was observed in the cpDNA region. In this study, in the *trn*S-*trn*G spacer, three and one segregating sites were found in *B. gymnorrhiza* and *B. sexangula*, respectively, and one fixed substitution was observed between the two species. Zhou et al. (2008) reported that no polymorphism was observed within the species and the two species differed by a single substitution. No polymorphism in the previous study by Zhou et al. (2008) is probably due to limited range of their samples. Sampling strategy is important for assessing genetic variability of widespread plants.

Two of the ten genes, *NAC* and *PO*, were monomorphic in the ten populations investigated in this study. In the *NAC* gene, nucleotide divergence between the species was comparable to the average divergence of the ten genes, suggesting that the gene is not so conservative. The weakly significant result in the multilocus HKA test probably reflects this inconsistency.

### Population structure and demographic history

The *F*_*ST*_ values among the ten populations were highly significant in both nuclear and cpDNA regions (Table 4), indicating that the ten populations were genetically differentiated. Average distance of pollen and propagule dispersal was short in *K. candel* (Geng et al. 2008). Like *K. candel*, *B. gymnorrhiza* is insect-pollinated and has viviparous propagules. Therefore, pollen and seed dispersal of *B. gymnorrhiza* appear to be limited. Consistent with this expectation, most pairwise *F*_*ST*_ values were significant.

The STRUCTURE analysis revealed that there are two possible genetic clusters. One cluster consists of Australian populations and populations located on the eastern side of Malay Peninsula. The other cluster includes populations on the western side of the Malay Peninsula as well as populations of Madagascar and India. The estimated migration rates between two clusters were low (2*Nm*_*E*_ = 0.29 and 2*Nm*_*W*_ = 0.04). As is the case of other mangrove species in the South East Asia (e.g., Liao et al. 2007; Inomata et al. 2009), the Sundaland and/or the Malay Peninsula prevented dispersion of *B. gymnorrhiza* and worked as a land barrier as well.

To obtain the splitting time in the actual demographic unit (in years), the estimated population splitting parameters need to be divided by the mutation rate per locus per year. As it is difficult to estimate mutation rate of *Bruguiera* species and unfortunately appropriate fossils are not available, the synonymous mutation rate estimated for other tree species was used; 0.7 × 10^−9^ per site per year in *Pinus* (Willyard et al. 2007) and 2.6 × 10^−9^ per site per year in palms (Gaut et al. 1996). We considered these rates as minimum and maximum mutation rates for *Bruguiera* species. Mutation rate per site per year for nonsynonymous sites was computed by multiplying synonymous mutation rate by the observed *K*_a_/*K*_*s*_ ratio (Table 3). If we assume *u* = 2.6 × 10^−9^ per site per year, the geometric mean mutation rate becomes 5.6 × 10^−7^ per locus per year. Point estimates of the splitting time between the two clusters in *B. gymnorrhiza* and the speciation time between *B. gymnorrhiza* and *B. sexangula* become 0.30 MYA and 0.57 MYA, respectively. If we assume *u* = 0.7 × 10^−9^ per site per year, the geometric mean mutation rate becomes 1.5 × 10^−7^ per locus per year. Point estimates of the splitting time and speciation time become 1.13 MYA and 2.12 MYA, respectively. The estimated splitting time of the two clusters is in the range from 0.30 to 1.13 MYA. The speciation time between *B. gymnorrhiza* and *B. sexangula* was in the range from 0.57 to 2.12 MYA. These estimates imply that the speciation of *B. gymnorrhiza* and *B. sexangula* happened in the middle Pleistocene, but the dispersion of *B. gymnorrhiza* did not happen until the late Pleistocene.

During the LGM, the sea level dropped ∼120 m compared with the present geographic area, and the Sundaland was formed in the Southeast Asia. At that time three localities examined in this study (MK, northwestern Borneo; ML, northwestern part of the Malay Peninsula and V, eastern part of Asian Continent) were not coast, while one of them (BMJ, eastern Java) was considered to be the edge of the Sundaland. Extant populations located on the previous Sundaland should be established after the LGM. Consistent with this, the BMJ (eastern Java) population showed higher polymorphism than the ML (northwestern part of the Malay Peninsula) and MK (northwestern Borneo) populations (Table S2). In addition, the BMJ (eastern Java) population harbors five of thirteen haplotypes in the *mang*-1 and a unique haplotype in the *SF*, *EXP*2, and *GM* (data not shown). Except for comparisons with the V (eastern part of the Asian Continent) population, pairwise *F*_*ST*_ values were much higher in comparisons with the BMJ (eastern Java) population (Table S4: BMJ – MK (northwestern Borneo): 0.69, BMJ (the east of the Java) – ML (the northwest of the Malay Peninsula): 0.68, MK (northwestern Borneo) – ML (northwestern part of the Malay Peninsula): 0.10). In the Southeast Asia the BMJ (eastern Java) population could be an ancient population compared with others.

## Data Accessibility

DNA sequences: DDBJ accessions AB813916-AB817042. IMa2 and STRUCTURE input files: the Dryad Repository: http://dx.doi.org/10.5061/dryad.bq858

## Supporting Information

**Figure S1.** Plot of delta *K* calculated using the method of Evanno et al. (2005).

**Figure S2.** Haplotype network of the cpDNA region. Circle size is approximately proportional to the number of individuals of each haplotype. Intermediate black dots indicate putative haplotypes. Solid and dotted lines indicate a single substitution and indels, respectively. Indel length is given above or below each dotted line.

**Figure S3.** Geographical distribution of the cpDNA haplotypes in *Bruguiera gymnorrhiza*. Circle size shows the number of individuals used in each population. Colors and patterns correspond to those of haplotype shown in Figure S2.

**Table S1.** PCR primers and annealing temperature.

**Table S2.** Nucleotide variation in *Bruguiera gymnorrhiza*.

**Table S3.** Summary of neutrality tests.

**Table S4.** Pairwise *F*_*ST*_ values (below diagonal) and *P*-values (above diagonal).
