# Supplementary figures and images for: Inferences of evolutionary history of a widely distributed mangrove species, Bruguiera gymnorrhiza, in the Indo-West Pacific region
Source: Ecol Evol. 2013 Jun 7;3(7):2251–61. doi: 10.1002/ece3.624 (PMC3728962; doi:10.1002/ece3.624)

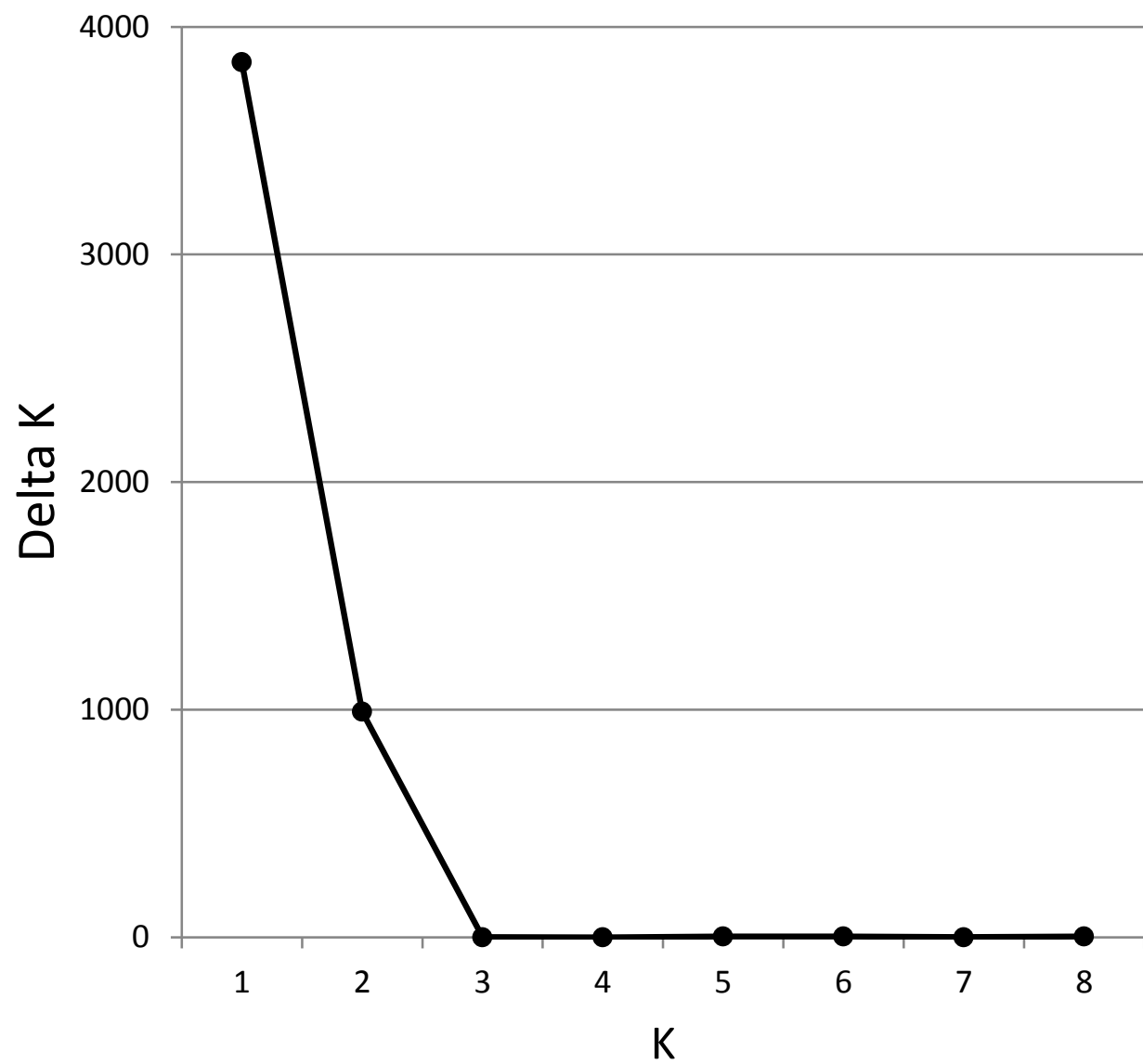

Supplement: Supplementary file 1 [file ece30003-2251-SD1.pdf]

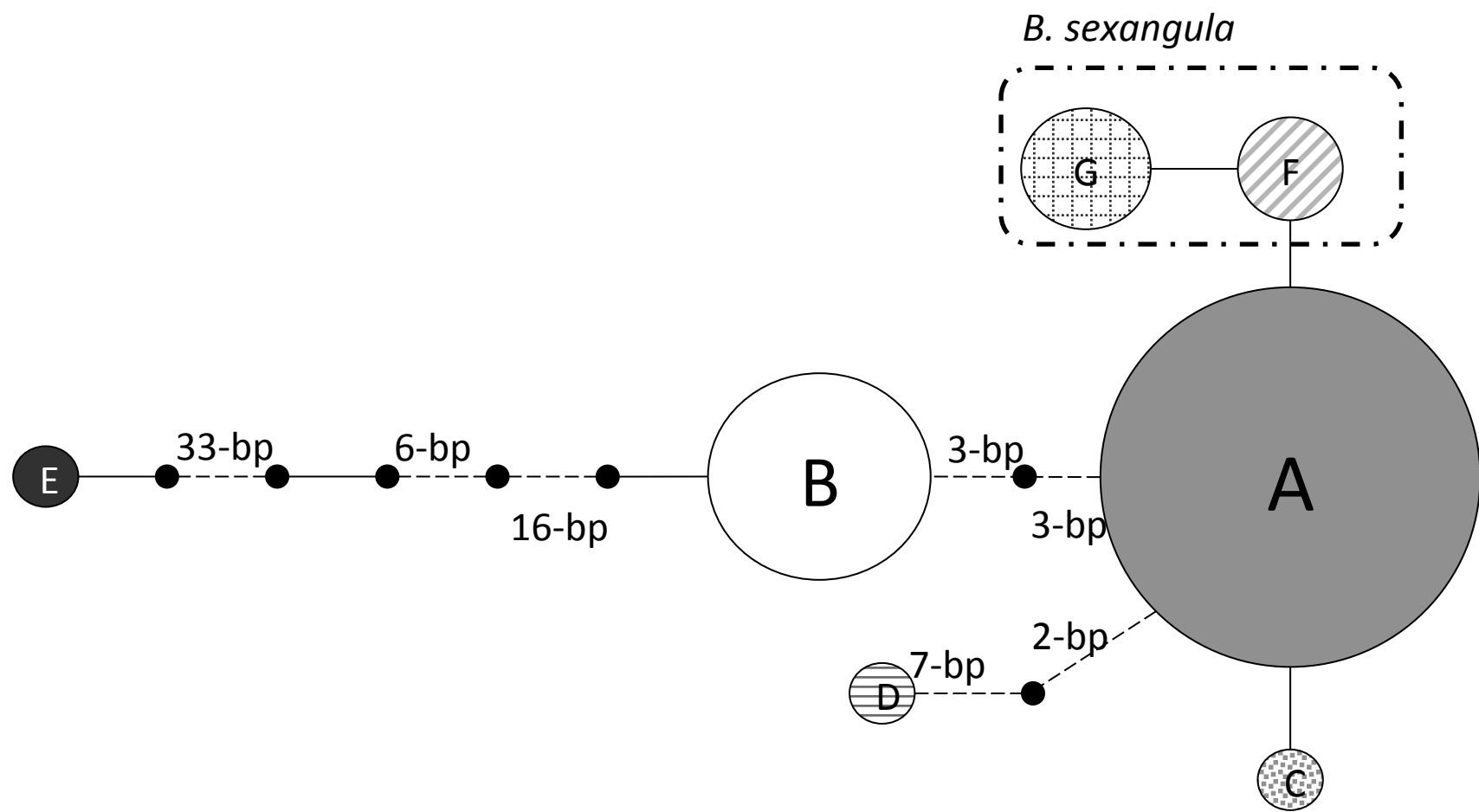

Supplement: Supplementary file 2 [file ece30003-2251-SD2.pdf]

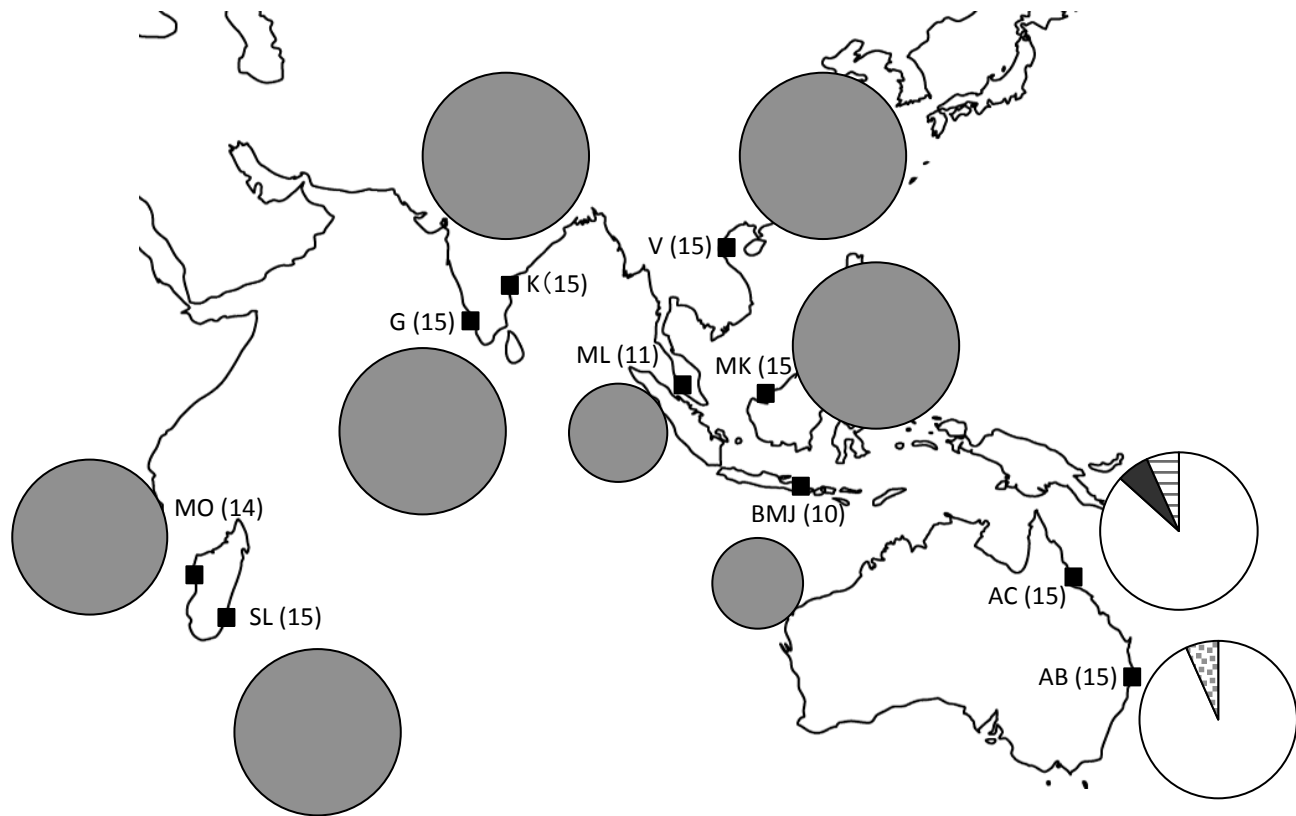

Supplement: Supplementary file 3 [file ece30003-2251-SD3.pdf]
