# Supplementary material for: Inferences of evolutionary history of a widely distributed mangrove species, Bruguiera gymnorrhiza, in the Indo-West Pacific region
Source: Ecol Evol. 2013 Jun 7;3(7):2251–61. doi: 10.1002/ece3.624 (PMC3728962; doi:10.1002/ece3.624)
Supplement: Supplementary file 5 [file ece30003-2251-SD5.doc]

Table S1. PCR primers and annealing temperature

| Locus | Primer name | Sequence (5'-3') | Annealing temperature |
| --- | --- | --- | --- |
| Nuclear genes |  |  |  |
| *NAC* | BP941102F | CGAGCTTCTTGATTTtTACCTCA | 55℃ |
|  | BP941102R | CGTTCATGACCCAATCAGTCT |
| *VVHP* | BP944085F | CCCAGAAACAAATGTAATGG | 55℃ |
|  | BP944085R | CCTTGCAGCAGCTGGAGT |
| *PO* | BP939063F | GATGCCATTAATGACCTTGGA | 55℃ |
|  | BP939063R | CCCATGACTGGCTGAAATAAA |
| *GM* | BP939421F | ACATCTGACCACAGGCCTTC | 55℃ |
|  | BP939421R | CACTAATATCATCAGGAGAGCC |
| *SF* | BP939928F | TGCTTAAGGCTCACAAGAAGG | 55℃ |
|  | BP939928R | GAGAATAACAGCTGCACCCAA |
| *EXP2* | BP942725F | GCCCACGCTACTTTCTACG | 60℃ |
|  | BP942725R | AGCGATTTTGAGGAACATGG |
| *UNK* | BP945250F | ACATGATGATCGGAGAACTTCC | 50℃ |
|  | BP945250R | CGGGTGTAGCCAGCACTC |
| *EPCRF* | BP946081F | AGCTGTCGTCTGAAATTCTGTCT | 50℃ |
|  | BP946081R | CAGATCAAGCTGGTATCGGAG |
| *PAL1* | PAL1-For | GAGCGCCAATTGGGTTGCTTT | 55℃ |
|  | PAL1-Rev | TGAGCAAACATGAGCTTTCCTAT |
| *mang-1* | manRev | GCCTTGGCCGCCGGCATCGGCT | 60℃ |
|  | manR2 | ATTATACAACAGAAAC |
|  | manFor3 | AGAGAGATCGTGGAAGCCCTGC |
| cpDNA region | |  |  |
| *trnS-trnG* | trnG-trnS.For | GAACGAATCACACTTTTACCAC | 60℃ |
|  | trnG-trnS.Rev | GCCGCTTTAGTCCACTCAGC |
| Cloning |  |  |  |
| T7 | T7 | AATACGACTCACTATAG | 55℃ |
| Reverse | Reverse | AACAGCTATGACCATG |

Table S2. Nucleotide variation in *B. gymnorrhiza*

| Population | Nuclear gene | | | cpDNA region | | |
| --- | --- | --- | --- | --- | --- | --- |
|  | *π_t_* | *π_s_* | *π_a_* | *π_t_* | *π_s_* | *π_a_* |
| AB | 0.55 | 0.68 | 0.00 | 0.26 | 0.26 | 0.00 |
| AC | 0.83 | 0.87 | 0.23 | 0.52 | 0.52 | 0.00 |
| BMJ | 1.46 | 2.26 | 0.05 | 0.00 | 0.00 | 0.00 |
| G | 0.00 | 0.00 | 0.00 | 0.00 | 0.00 | 0.00 |
| K | 0.00 | 0.00 | 0.00 | 0.00 | 0.00 | 0.00 |
| MK | 0.01 | 0.00 | 0.04 | 0.00 | 0.00 | 0.00 |
| ML | 0.10 | 0.17 | 0.09 | 0.00 | 0.00 | 0.00 |
| MO | 1.35 | 1.84 | 0.59 | 0.00 | 0.00 | 0.00 |
| SL | 0.11 | 0.20 | 0.06 | 0.00 | 0.00 | 0.00 |
| V | 2.12 | 4.24 | 0.47 | 0.00 | 0.00 | 0.00 |
| Total | 4.11 | 8.00 | 0.55 | 0.08 | 0.08 | 0.00 |

Total, when ten populations were regarded as a single population.

*π_t_*, Number of nucleotide differences per total site (Nucleotide diversity; Nei 1987) with the Jukes and Cantor correction (1969). Indels are not included. The value was multiplied by 10^3^.

*π_s_*, Number of nucleotide differences per silent site with the Jukes and Cantor correction (1969). Indels are not included. The value was multiplied by 10^3^.

*π_a_*, Number of nucleotide differences per nonsynonymous site with the Jukes and Cantor correction (1969). Indels are not included. The value was multiplied by 10^3^.

Table S3. Summary of neutrality tests

| Nuclear gene | population | Tajima's *D* | Fu & Li's *D* | Fu & Li's *F* | Fay & Fu's *H* |
| --- | --- | --- | --- | --- | --- |
| NAC | AB | n.a. | n.a. | n.a. | n.a. |
|  | AC | n.a. | n.a. | n.a. | n.a. |
|  | BMJ | n.a. | n.a. | n.a. | n.a. |
|  | G | n.a. | n.a. | n.a. | n.a. |
|  | K | n.a. | n.a. | n.a. | n.a. |
|  | MK | n.a. | n.a. | n.a. | n.a. |
|  | ML | n.a. | n.a. | n.a. | n.a. |
|  | MO | n.a. | n.a. | n.a. | n.a. |
|  | SL | n.a. | n.a. | n.a. | n.a. |
|  | V | n.a. | n.a. | n.a. | n.a. |
|  | Total | n.a. | n.a. | n.a. | n.a. |
| VVHP | AB | -1.507 | -2.281 | -2.382 | 0.129 |
|  | AC | -1.147 | -1.682 | -1.766 | 0.064 |
|  | BMJ | n.a. | n.a. | n.a. | n.a. |
|  | G | n.a. | n.a. | n.a. | n.a. |
|  | K | n.a. | n.a. | n.a. | n.a. |
|  | MK | -1.147 | -1.682 | -1.766 | 0.064 |
|  | ML | n.a. | n.a. | n.a. | n.a. |
|  | MO | 3.340** | 1.468* | 2.389* | 0.460 |
|  | SL | n.a. | n.a. | n.a. | n.a. |
|  | V | 0.102 | -1.951 | -1.524 | 0.772 |
|  | Total | 0.552 | -4.472* | -2.876* | 1.715 |
| PO | AB | n.a. | n.a. | n.a. | n.a. |
|  | AC | n.a. | n.a. | n.a. | n.a. |
|  | BMJ | n.a. | n.a. | n.a. | n.a. |
|  | G | n.a. | n.a. | n.a. | n.a. |
|  | K | n.a. | n.a. | n.a. | n.a. |
|  | MK | n.a. | n.a. | n.a. | n.a. |
|  | ML | n.a. | n.a. | n.a. | n.a. |
|  | MO | n.a. | n.a. | n.a. | n.a. |
|  | SL | n.a. | n.a. | n.a. | n.a. |
|  | V | n.a. | n.a. | n.a. | n.a. |
|  | Total | n.a. | n.a. | n.a. | n.a. |
| GM | AB | 1.621 | 0.594 | 1.010 | -0.074 |
|  | AC | 1.635 | 0.594 | 1.015 | 0.000 |
|  | BMJ | -1.164 | -1.540 | -1.648 | 0.095 |
|  | G | n.a. | n.a. | n.a. | n.a. |
|  | K | n.a. | n.a. | n.a. | n.a. |
|  | MK | n.a. | n.a. | n.a. | n.a. |
|  | ML | -1.162 | -1.575 | -1.678 | 0.087 |
|  | MO | n.a. | n.a. | n.a. | n.a. |
|  | SL | n.a. | n.a. | n.a. | n.a. |
|  | V | 0.395 | 1.214 | 1.129 | 0.000 |
|  | Total | 2.668* | -0.468 | 0.806 | 0.036 |
| SF | AB | n.a. | n.a. | n.a. | n.a. |
|  | AC | n.a. | n.a. | n.a. | n.a. |
|  | BMJ | 0.611 | 0.866 | 0.915 | -0.368 |
|  | G | n.a. | n.a. | n.a. | n.a. |
|  | K | n.a. | n.a. | n.a. | n.a. |
|  | MK | n.a. | n.a. | n.a. | n.a. |
|  | ML | -0.641 | 0.635 | 0.336 | -1.558 |
|  | MO | n.a. | n.a. | n.a. | n.a. |
|  | SL | n.a. | n.a. | n.a. | n.a. |
|  | V | 1.477 | 0.806 | 1.150 | -0.331 |
|  | Total | 1.254 | 0.750 | 1.088 | 0.218 |
| EXP2 | AB | 0.819 | 0.603 | 0.761 | -0.778 |
|  | AC | -0.057 | 1.543* | 0.926 | -7.347 |
|  | BMJ | 1.478 | 1.518* | 1.749* | -2.632 |
|  | G | n.a. | n.a. | n.a. | n.a. |
|  | K | n.a. | n.a. | n.a. | n.a. |
|  | MK | n.a. | n.a. | n.a. | n.a. |
|  | ML | n.a. | n.a. | n.a. | n.a. |
|  | MO | 1.343 | 0.815 | 1.111 | -0.016 |
|  | SL | n.a. | n.a. | n.a. | n.a. |
|  | V | 0.940 | 0.594 | 0.794 | -0.708 |
|  | Total | -0.710 | 1.669* | 2.884* | -0.792 |
| UNK | AB | n.a. | n.a. | n.a. | n.a. |
|  | AC | 1.507 | 0.594 | 0.974 | 0.166 |
|  | BMJ | n.a. | n.a. | n.a. | n.a. |
|  | G | n.a. | n.a. | n.a. | n.a. |
|  | K | n.a. | n.a. | n.a. | n.a. |
|  | MK | n.a. | n.a. | n.a. | n.a. |
|  | ML | n.a. | n.a. | n.a. | n.a. |
|  | MO | n.a. | n.a. | n.a. | n.a. |
|  | SL | -0.409 | 0.594 | 0.365 | 0.166 |
|  | V | 1.280 | 0.594 | 0.902 | -0.460 |
|  | Total | 0.464 | 0.616 | 0.672 | 0.265 |
| EPCRF | AB | n.a. | n.a. | n.a. | n.a. |
|  | AC | -1.507 | -2.281 | -2.382 | 0.129 |
|  | BMJ | n.a. | n.a. | n.a. | n.a. |
|  | G | n.a. | n.a. | n.a. | n.a. |
|  | K | n.a. | n.a. | n.a. | n.a. |
|  | MK | n.a. | n.a. | n.a. | n.a. |
|  | ML | n.a. | n.a. | n.a. | n.a. |
|  | MO | n.a. | n.a. | n.a. | n.a. |
|  | SL | n.a. | n.a. | n.a. | n.a. |
|  | V | -0.383 | 1.144 | 0.806 | -2.106 |
|  | Total | 1.640 | -0.856 | 0.032 | 0.025 |
| PAL1 | AB | n.a. | n.a. | n.a. | n.a. |
|  | AC | n.a. | n.a. | n.a. | n.a. |
|  | BMJ | n.a. | n.a. | n.a. | n.a. |
|  | G | n.a. | n.a. | n.a. | n.a. |
|  | K | n.a. | n.a. | n.a. | n.a. |
|  | MK | n.a. | n.a. | n.a. | n.a. |
|  | ML | -0.1747 | 0.635 | 0.479 | 0.208 |
|  | MO | 1.213 | 0.603 | 0.885 | 0.238 |
|  | SL | 0.216 | 0.594 | 0.564 | -1.149 |
|  | V | n.a. | n.a. | n.a. | n.a. |
|  | Total | 0.705 | 0.860 | 0.962 | 0.697 |
| *mang*-1 | AB | 1.323 | 0.690 | 1.030 | -1.287 |
|  | AC | -1.732 | -2.688* | -2.794* | -1.738 |
|  | BMJ | 0.887 | -0.124 | 0.175 | -1.958 |
|  | G | n.a. | n.a. | n.a. | n.a. |
|  | K | n.a. | n.a. | n.a. | n.a. |
|  | MK | n.a. | n.a. | n.a. | n.a. |
|  | ML | n.a. | n.a. | n.a. | n.a. |
|  | MO | -0.019 | 0.603 | 0.497 | 0.212 |
|  | SL | -1.732 | -2.688* | -2.794* | -1.738 |
|  | V | 2.130* | 0.806 | 1.363 | 0.129 |
|  | Total | -0.009 | -0.368 | -0.276 | 0.337 |
| cpDNA |  |  |  |  |  |
| trnSG | AB | -1.159 | -1.427 | -1.543 | 0.124 |
|  | AC | -1.491 | -1.873 | -1.873 | 0.248 |
|  | BMJ | n.a. | n.a. | n.a. | n.a. |
|  | G | n.a. | n.a. | n.a. | n.a. |
|  | K | n.a. | n.a. | n.a. | n.a. |
|  | MK | n.a. | n.a. | n.a. | n.a. |
|  | ML | n.a. | n.a. | n.a. | n.a. |
|  | MO | n.a. | n.a. | n.a. | n.a. |
|  | SL | n.a. | n.a. | n.a. | n.a. |
|  | V | n.a. | n.a. | n.a. | n.a. |
|  | Total | -1.560 | -3.578* | -3.452* | 0.043 |

*, *P* < 0.05

**, *P* < 0.001

n.a., Not applicable.

Table S4. Pairwise *F_ST_* values (below diagonal) and *P*-values (above diagonal)

|  | AB | AC | BMJ | V | MK | ML | K | G | MO | SL |
| --- | --- | --- | --- | --- | --- | --- | --- | --- | --- | --- |
| AB |  | ** | ** | ** | NS | NS | ** | ** | ** | ** |
| AC | 0.11384 |  | ** | ** | ** | NS | ** | ** | ** | ** |
| BMJ | 0.69100 | 0.66901 |  | ** | ** | ** | ** | ** | NS | ** |
| V | 0.88379 | 0.89080 | 0.91222 |  | ** | ** | NS | ** | ** | ** |
| MK | -0.03448 | 0.11384 | 0.69100 | 0.88379 |  | NS | ** | ** | ** | ** |
| ML | 0.10005 | -0.03291 | 0.67820 | 0.91199 | 0.10005 |  | ** | ** | ** | ** |
| K | 0.88379 | 0.89080 | 0.91222 | 0.00000 | 0.88379 | 0.91199 |  | ** | ** | ** |
| G | 0.88379 | 0.89080 | 0.91278 | 1.00000 | 0.88379 | 0.91199 | 1.00000 |  | ** | NS |
| MO | 0.46752 | 0.42072 | 0.06128 | 0.80611 | 0.46752 | 0.41962 | 0.80611 | 0.80700 |  | ** |
| SL | 0.88379 | 0.89080 | 0.91278 | 1.00000 | 0.88379 | 0.91199 | 1.00000 | 0.00000 | 0.80700 |  |

NS, Not significant

**, *P* < 0.01 after the Bonferroni correction.
