# Supplementary material for: Inferences of evolutionary history of a widely distributed mangrove species, Bruguiera gymnorrhiza, in the Indo-West Pacific region
Source: Ecol Evol. 2013 Jun 7;3(7):2251–61. doi: 10.1002/ece3.624 (PMC3728962; doi:10.1002/ece3.624)
Supplement: Supplementary file 6 [file ece30003-2251-SD6.doc]

Data Accessibility

DNA sequences: DDBJ accessions AB813916-AB817042.

IMa2 and STRUCTURE input files: the Dryad Repository: <http://dx.doi.org/>10.5061/dryad.bq858

Supporting Information

Fig. S1 Plot of delta *K* calculated using the method of Evanno et al. (2005).

Fig. S2 Haplotype network of the cpDNA region.

Fig. S3 Geographic distribution of the cpDNA haplotypes in *B*. *gymnorrhiza*.

Table S1 List of PCR primers and annealing temperature.

Table S2 Nucleotide variation in each population of *B. gymnorrhiza*

Table S3 Summary of neutrality tests

Table S4 Pairwise *F_ST_* values (below diagonal) and *P*-values (above diagonal)
